# A New Triterpenoid Glucoside from a Novel Acidic Glycosylation of Ganoderic Acid A via Recombinant Glycosyltransferase of *Bacillus subtilis*

**DOI:** 10.3390/molecules24193457

**Published:** 2019-09-24

**Authors:** Te-Sheng Chang, Chien-Min Chiang, Yu-Han Kao, Jiumn-Yih Wu, Yu-Wei Wu, Tzi-Yuan Wang

**Affiliations:** 1 Department of Biological Sciences and Technology, National University of Tainan, Tainan 70005, Taiwan; mozyme2001@gmail.com (T.-S.C.); aa0920281529@gmail.com (Y.-H.K.); 2 Department of Biotechnology, Chia Nan University of Pharmacy and Science, No. 60, Sec. 1, Erh-Jen Rd., Jen-Te District, Tainan 71710, Taiwan; cmchiang@mail.cnu.edu.tw; 3 Department of Food Science, National Quemoy University, Kinmen County 892, Taiwan; wujy@nqu.edu.tw; 4 Graduate Institute of Biomedical Informatics, College of Medical Science and Technology, Taipei Medical University, Taipei 106, Taiwan; yuwei.wu@tmu.edu.tw; 5 Clinical Big Data Research Center, Taipei Medical University Hospital, Taipei 110, Taiwan; 6 Biodiversity Research Center, Academia Sinica, Taipei 115, Taiwan

**Keywords:** ganoderic acid A, glucosyltransferase, acidic, *Bacillus subtilis*, triterpenoid

## Abstract

Ganoderic acid A (GAA) is a bioactive triterpenoid isolated from the medicinal fungus *Ganoderma lucidum*. Our previous study showed that the *Bacillus subtilis* ATCC (American type culture collection) 6633 strain could biotransform GAA into compound (**1**), GAA-15-*O*-β-glucoside, and compound (**2**). Even though we identified two glycosyltransferases (GT) to catalyze the synthesis of GAA-15-*O*-β-glucoside, the chemical structure of compound (**2**) and its corresponding enzyme remain elusive. In the present study, we identified BsGT110, a GT from the same *B. subtilis* strain, for the biotransformation of GAA into compound (**2**) through acidic glycosylation. BsGT110 showed an optimal glycosylation activity toward GAA at pH 6 but lost most of its activity at pH 8. Through a scaled-up production, compound (**2**) was successfully isolated using preparative high-performance liquid chromatography and identified to be a new triterpenoid glucoside (GAA-26-*O*-β-glucoside) by mass and nuclear magnetic resonance spectroscopy. The results of kinetic experiments showed that the turnover number (k_cat_) of BsGT110 toward GAA at pH 6 (k_cat_ = 11.2 min^−1^) was 3-fold higher than that at pH 7 (k_cat_ = 3.8 min^−1^), indicating that the glycosylation activity of BsGT110 toward GAA was more active at acidic pH 6. In short, we determined that BsGT110 is a unique GT that plays a role in the glycosylation of triterpenoid at the C-26 position under acidic conditions, but loses most of this activity under alkaline ones, suggesting that acidic solutions may enhance the catalytic activity of this and similar types of GTs toward triterpenoids.

## 1. Introduction

*Ganoderma lucidum* is a medicinal fungus that has been used to improve health and prevent certain diseases in Asia for thousands of years [1]. In modern ages, many bioactive compounds such as polysaccharides and triterpenoids [2,3] were identified and isolated from *G. lucidum*. These compounds were demonstrated to be effective for anti-cancer, anti-oxidant, anti-bacterial, anti-inflammation, and immune-regulation purposes [2,3].

Unlike triterpenoids from ginseng plants—which usually exist in the glycosidic form, called ginseng saponins—very few *Ganoderma* triterpenoid glycosides have been identified, despite the existence of many triterpenoids in *G. lucidum* [4]. The glycosidic form of triterpenoids might improve the bioactivity of the triterpenoids. For example, several ginseng saponins were found to exhibit more bioactivities involved in the central nervous system, cardiovascular system, and immune functions than ginseng triterpenoid aglycones were [5]. The glycosylation of flavonoids can also increase both water solubility and flavonoid stability [6,7,8]. It is, therefore, worthwhile to investigate the glycosylation of *Ganoderma* triterpenoids for potential medical and clinical purposes.

In nature, glycosylation is usually catalyzed by glycosyltransferase (GT, EC 2.4.x.y), a type of enzyme that uses a nucleotide-activated sugar donor, such as uridine diphosphate (UDP)-glucose, to transfer the sugar moiety to a sugar acceptor molecule [9]. Several GTs that catalyze the glycosylation of triterpenoids have already been discovered from plants, due to the accumulating knowledge on the metabolic pathways of triterpenoid glycosides [10]. However, plant GTs are not good candidates for the biotransformation of xenobiotics (such as *Ganoderma* triterpenoids) because plant GTs are usually involved in triterpenoid biosynthesis pathways and thus have very high substrate specificity. In contrast, GTs from bacterial sources usually have lower substrate specificity and have been demonstrated to be involved in the glycosylation of ginseng triterpenoids [11].

**Figure 1:**
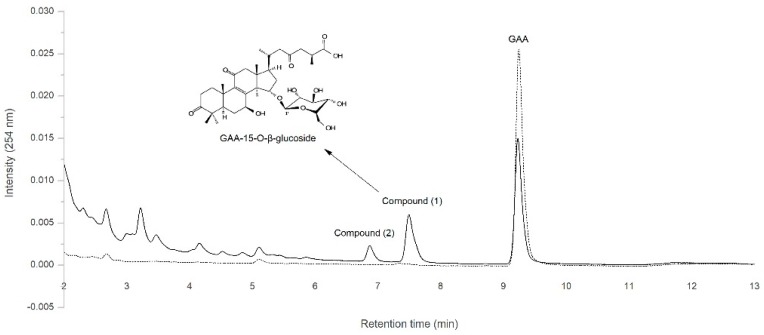
Biotransformation of ganoderic acid A (GAA) by *Bacillus subtilis* ATCC 6633 after 24 h of incubation (solid line). The figure was modified from Figure 1 of our previous study [17].

Among the *Ganoderma lucidum* bioactive compounds, ganoderic acid A (GAA) is one of the major triterpenoids and has been shown to prevent the proliferation of cancer cells and reduce inflammation activities [12,13,14,15,16]. Our previous study showed that the *Bacillus subtilis* ATCC (American type culture collection) 6633 strain can biotransform GAA into one major compound (**1**) and one minor compound (**2**) (Figure 1) [17]. In addition, two GTs—BsUGT398 and BsUGT489—were identified to catalyze the biotransformation of GAA into compound (**1**), which was later identified as GAA-15-*O*-β-glucoside [17]. However, the chemical structure of the compound (**2**) and its corresponding catalyzing enzyme remain elusive. In the present study, a GT enzyme that catalyzes the biotransformation of GAA to compound (**2**) was successfully identified, along with the optimal condition for producing compound (**2**) by the GT enzyme. The chemical structure of the previously-unknown compound (**2**) was also elucidated with the scaled-up production of the GT enzyme under an acidic condition.

## 2. Results

### 2.1. Biotransformation of GAA by Recombinant BsGT110 from B. subtilis ATCC 6633

**Figure 2:**
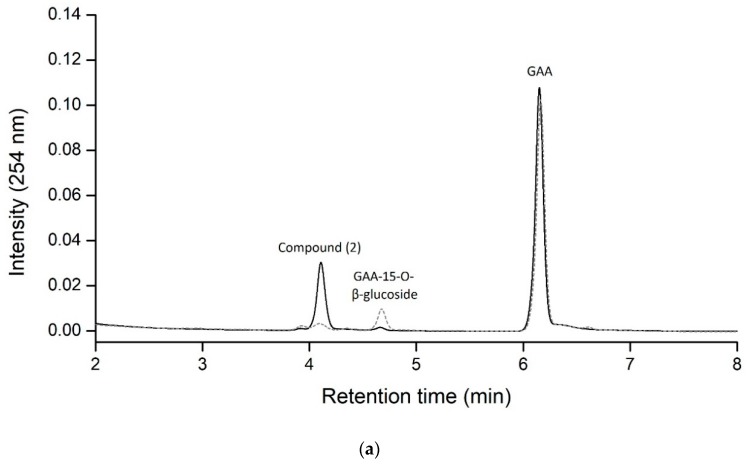

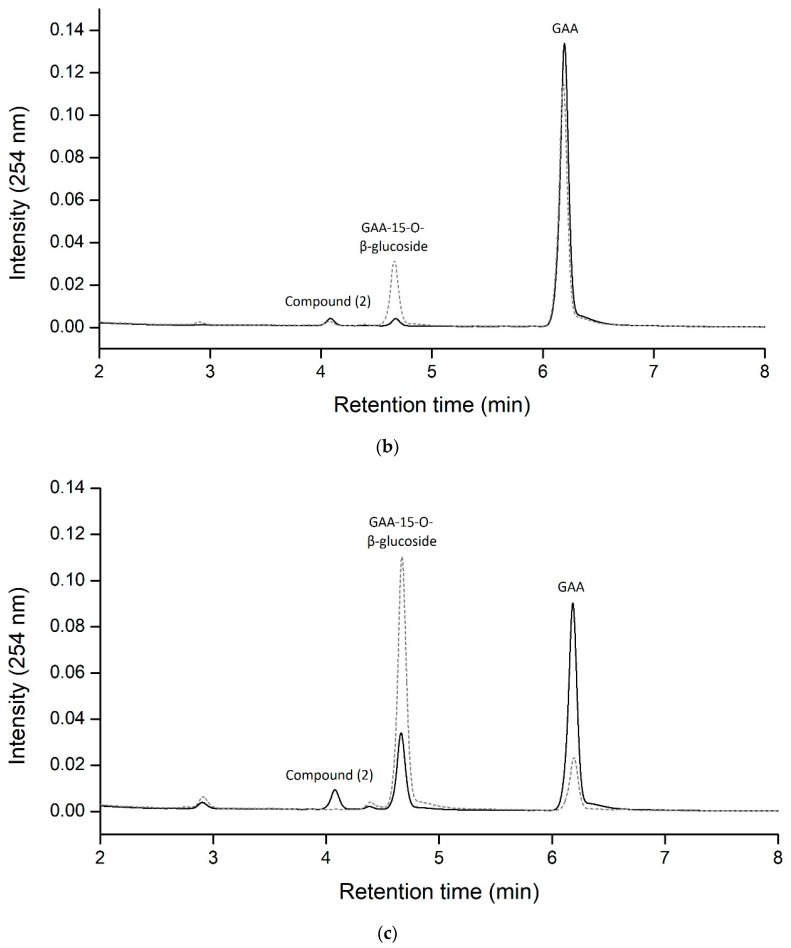
Ultra-performance liquid chromatography (UPLC) analysis of the biotransformation of GAA by BsGT110 (**a**), BsUGT398 (**b**), and BsUGT489 (**c**) at pH 6 (solid line) and pH 8 (dashed line). The biotransformation mixture contained 15 μg/mL purified enzyme, 1 mg/mL GAA, 10 mM uridine diphosphate (UDP)-glucose, 10 mM MgCl_2_, and 50 mM acetate buffer at pH 6 or phosphate buffer (PB) at pH 8 and was incubated at 40 °C for 30 min. After incubation, the reaction was analyzed using UPLC. The UPLC operation procedure was described in the Materials and Methods section.

Our previous study showed that *B. subtilis* ATCC 6633 can biotransform GAA primarily into one major compound (**1**), GAA-15-*O*-β-glucoside, and one unknown minor compound (**2**) (Figure 1) [17]. To obtain enough unknown compound (**2**) through in vitro enzymatic biotransformation and then identify that compound’s chemical structure, we strived to identify corresponding GT enzymes from the *B. subtilis* ATCC 6633 strain. In our previous work, we selected five GT genes—BsGT110, BsGT292, BsGT296, BsUGT398, and BsUGT489—and successfully overexpressed and purified them in *Escherichia coli* [17]. However, none of them were found to catalyze the biotransformation of GAA into compound (**2**) under a general GT reaction condition: 10 mM Mg^2+^, 40 °C, and pH 8 [17]. We assayed the five recombinant BsGTs under different pH values and determined that BsGT110 produces a reasonable amount of compound (**2**) from the biotransformation of GAA under an acidic condition (pH 6), as shown in the solid line in Figure 2a. BsUGT398 and BsUGT489 produced only small amounts of compound (**2**) under the acidic condition (pH 6) (solid lines in Figure 2b,c). As expected, compound (**2**) was no longer produced from the biotransformation of GAA by any of the three GTs at pH 8 (dashed lines in Figure 2a–c). BsUGT398 and BsUGT489 produced large amounts of GAA-15-*O*-β-glucoside at pH 8. However, no metabolite was detected from the reactions with BsGT292 and BsGT296 at pH 6 or pH 8 (data not shown). We thus selected BsGT110 to produce compound (**2**) at pH 6 for further analysis. The amount of GAA-15-*O*-β-glucoside and compound (**2**) that can be catalyzed from GAA by BsGT110 at different pH values were indicated in Table 1. It is noted that the maximum amount of compound (**2**) was produced under 1 mg/mL GAA, 10 mM UDP-glucose, 15 μg/mL BsGT110, 10 mM MgCl_2_, and 50 mM acetate buffer at pH 6.

**Figure 3:**
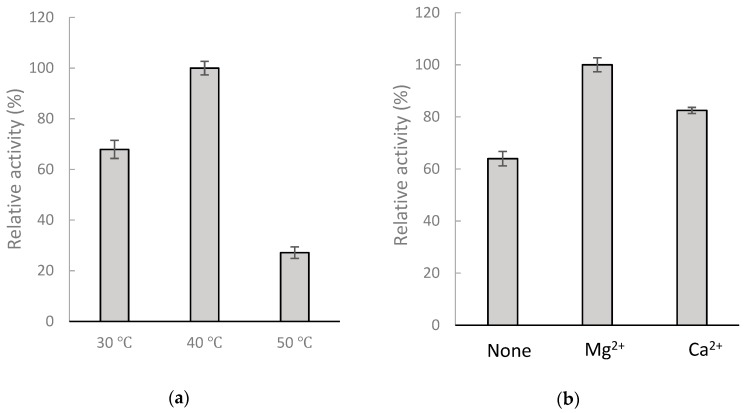
The production of compound (**2**) from GAA by BsGT110 under different temperature or metal ion conditions. The standard condition was set as 15 μg/mL purified enzyme, 1 mg/mL GAA, 10 mM MgCl_2_, and 10 mM UDP-glucose in 50 mM acetate buffer at pH 6.0 and 40 °C. The tests were carried out by changing only the temperature (**a**) or metal ions (**b**) and maintaining all other settings. Relative activities were obtained by dividing the area summation of the UPLC reaction peak of the test condition by that of the standard condition. The data are expressed as mean ± SD, *n* = 3.

To optimize the production of compound (**2**), a standard mixture was made of 1 mg/mL GAA, 10 mM UDP-glucose, 15 μg/mL BsGT110, and 50 mM acetate buffer at pH 6 under different temperature and metal ion conditions. After incubation, the amount of compound (**2**) produced was determined with UPLC (Figure 3). The results revealed that the optimal conditions for the production of compound (**2**) from GAA by the recombinant BsGT110 is pH 6, 40 °C, and 10 mM MgCl_2_. The relative production of GAA-15-*O*-β-glucoside was less than 5% for all testing conditions.

### 2.2. Identification of the Biotransformation Product

**Figure 4:**
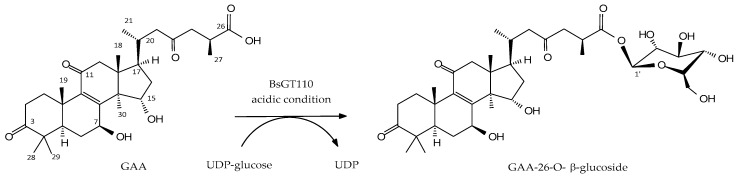
Biotransformation of GAA by BsGT110 in the acidic condition.

To resolve the chemical structure of compound (**2**), the biotransformation was scaled up to 25 mL, with 1 mg/mL GAA, 15 μg/mL BsGT110, 10 mM MgCl_2_, and 10 mM UDP-glucose in 50 mM acetate buffer of pH 6 and 40 °C for a 30-min incubation. A total of 5.4 mg of compound (**2**) in the 25-mL reaction was purified with preparative high-performance liquid chromatography (HPLC). The chemical structure of the purified compound was then resolved using mass and nucleic magnetic resonance (NMR) spectral analyses. The molecular formula of compound (**2**) was established as C_36_H_53_O_12_ by the electrospray ionization mass (ESI-MS) at m/z 679.67 [M + H]^+^, indicating the presence of a glucose residue. The NMR spectra exhibit characteristic glucosyl signals: the anomeric carbon signal at δ_C_ 95.9, one CH_2_ signal at δ_C_ 61.8, and four CH signals at δ_C_ 70.6, 73.9, 78.2, 79.1. The large coupling constant (8.1 Hz) of the anomeric proton H-1′ (6.33 ppm) indicated the β-configuration. The cross peak of H-1′ with C-26 (6.33/174.6 ppm) in the HMBC spectrum demonstrated the structure of compound (**2**) to be GAA-26-*O*-β-glucoside. The NMR spectra data are shown in Table S1 and Figures S1–S4. Figure 4 illustrated the biotransformation process of GAA by BsGT110.

### 2.3. Kinetic Study of the Biotransformation of GAA by BsGT110

**Figure 5:**
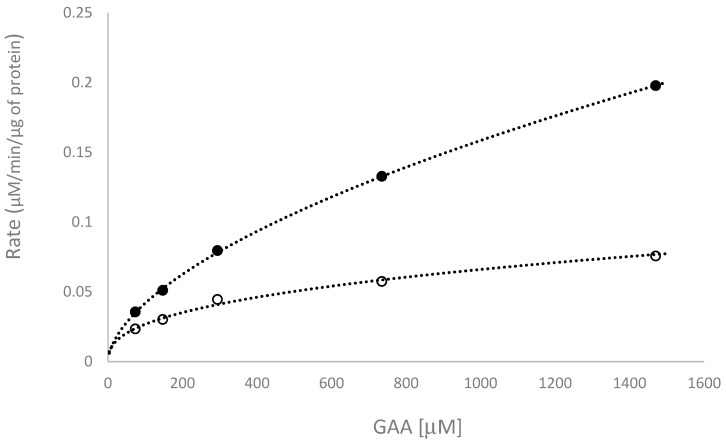
Kinetic study of BsGT110 at pH 6 (closed symbols) and pH 7 (open symbols). Different concentrations of GAA were mixed with 15 μg purified BsGT110 protein, 10 mM UDP-glucose, 10 mM MgCl_2_, and 50 mM PB (pH 7.0) or acetate buffer (pH 6) in 1 mL reaction mixture and incubated at 40 °C for 20 min. During the incubation, samples from each reaction were removed and analyzed by UPLC every 2 min. The reaction rate for each concentration of GAA was obtained from the slope of the plot of the amount of product over time. The UPLC operation procedure was described in the Materials and Methods section.

To study how pH affects the biotransformation activity of GAA by BsGT110, a kinetic study of the biotransformation was conducted at different concentrations of GAA, with 50 mM acetate buffer at pH 6 or PB at pH 7, 10 mM MgCl_2_, and 10 mM UDP-glucose, at 40 °C. The results of the kinetic study were shown in Figure 5, and the calculated kinetic parameters were listed in Table 2. The results showed that the turnover number (k_cat_) of BsGT110 toward GAA at pH 6 was 3-fold higher than that at pH 7.

## 3. Discussion

**Figure 6:**
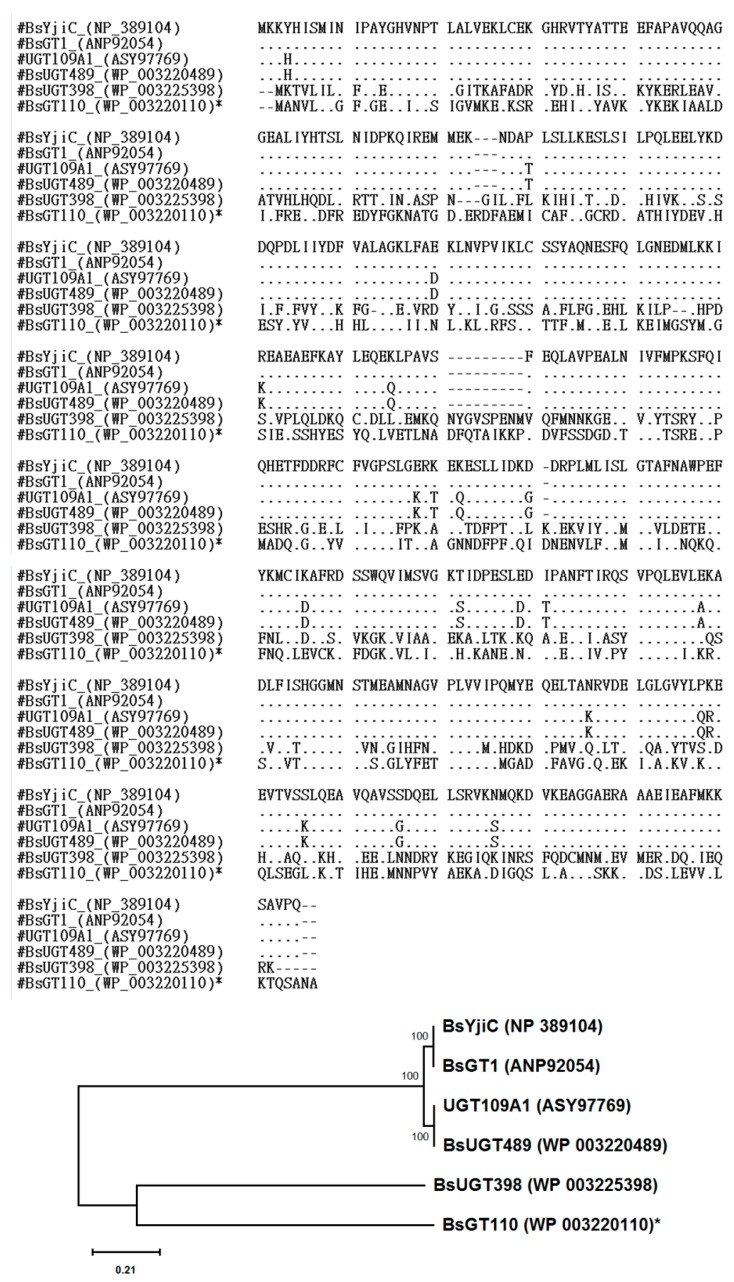
Aligned amino acid sequences and phylogenetic analysis using the Maximum Likelihood method. In total, 407 amino acids were aligned by Clustal W in MEGA X [27]. ‘.’ denoted as identical amino acid, ‘-’ denoted as indel(s). The phylogenetic tree was inferred using the Maximum Likelihood method and General Reversible Mitochondrial model [28]. The tree with the highest log likelihood (−3197.28) was shown. The percentage of trees in which the associated taxa clustered together was shown next to the branches. Initial tree(s) for the heuristic search were obtained automatically by applying Neighbor-Joining and BioNJ algorithms to a matrix of pairwise distances estimated using the JTT model, the topology with highest log-likelihood value was then selected. The tree was drawn to scale, with branch lengths measured based on the number of substitutions per site. This analysis involved six amino acid sequences. All positions with less than 95% site coverage were eliminated—i.e., less than 5% alignment gaps, missing data, and ambiguous bases were allowed at any position (partial deletion option). There were a total of 382 positions in the final dataset. Evolutionary analyses were conducted in MEGA X [27].

According to the Carbohydrate-Active Enzymes (CAZy) database, GTs can be classified into 107 families, in which GTs that catalyze the glycosylation of small molecules, such as flavonoids and triterpenoids, are classified as GT1 [18]. Although over 500 thousands of GT have been discovered, there are only six bacterial GTs reported to catalyze glycosylation of triterpenoids, including BsYjiC from *B. subtilis* 168 [11,19,20,21,22], UGT109A1 from *B. subtilis* CTCG 63501 [23,24], BsGT1 from *B. subtilis* KCTC 1022 [25], BsUGT398 and BsUGT489 from *B. subtilis* ATCC 6633 [17], and BsGT110 from *B. subtilis* ATCC 6633 [present study]. Among them, the BsYjiC group (BsYjiC, BsUGT489, UGT109A1, and BsGT1) were highly similar, sharing more than 90% identity in their amino acid sequences [17], BsGT110 and BsUGT398, however, were not grouped with the BsYjiC group, and only had 31% and 33% identity, respectively, with the BsYjiC group (Figure 6). On the other hand, some bacterial GT1 catalyzed glycosylation of flavonoids. Thus, BsGT110 was compared with the flavonoid-catalyzing GTs. The results showed that the amino acid identity between BsGT110 and other flavonoid-catalyzing bacterial GTs was also lower than 40% (Figure S5 and Table S2). Furthermore, the evolutionary tree is shown in Figure 6 also demonstrated the dissimilarity between BsGT110 and BsUGT398, indicating that BsGT110 is a unique bacterial GT with glycosylation activity toward triterpenoids.

There are two reaction mechanisms for GT, inverting and retaining reactions, depending on the outcome of the reaction [26]. There are two stereochemical outcomes for reactions that result in the formation of a new glycosidic bond: the anomeric configuration of the product is either retained or inverted with respect to the donor substrate. The mechanistic strategy for inverting GTs involves a side chain of a residue on the active-site of GT that serves as a base catalyst that deprotonates the incoming nucleophile of the acceptor, facilitating direct displacement of the activated (substituted) phosphate leaving the group of the sugar donor, UDP-glucose [26]. Up to now, all GT1s are inverting GTs and were not reported to show optimal activities in acidic conditions [26]. For example, the well-known triterpenoid-catalyzing BsGT1 [25] had optimal activity at pH 7, BsYjiC [20], BsUGT398, and BsUGT489 [17] had optimal activity at pH 8, and UGT109A1 [23] had optimal activity at pH 9–10. These triterpenoid-catalyzing GTs had a broad neutral-alkaline range in their triterpenoid glycosylation activity [17,20,21,22,23,24,25]. According to the reaction mechanism of the inverting GTs, the side-chain of a key residue in the catalytic site of the enzyme should be deprotonated to serve as a base during the reaction. Thus, it is reasonable that GT1 enzymes have optimal activities at neutral-alkaline conditions, which would favor the deprotonation of the side chain. Accordingly, we identified the glycosylation activity of the selected five BsGTs toward GAA under a standard GT reaction condition: 10 mM Mg^2+^, 40 °C, and pH 8, and found that only BsUGT398 and BsUGT489 can catalyze C-15 glycosylation of GAA [17], but other candidates, including BsGT110, were unable to catalyze glycosylation of GAA under the standard GT reaction condition. Thus, the previous study did not observe the novel acidic glycosylation activity (C-26 glycosylation of GAA) of BsGT110 toward GAA. Hence, the BsGT110 that we identified in this work was much more capable of catalyzing GAA into pure triterpenoid glucoside (GAA-26-*O*-β-glucoside) under acidic conditions (pH 5–6) (Figure 2 and Figure 4, and Table 1). In addition, the results of the kinetics study showed that the turnover number of BsGT110 toward GAA at pH 6 was 3-fold higher than that at pH 7 (Figure 5 and Table 2). Furthermore, the catalytic efficiency (k_cat_/K_m_) of BsGT110 toward GAA at pH 6 was 1.35-fold higher than that at pH 7. Taken together, our results are unique in that they indicate that BsGT110—unlike other GT1s, which are most active at regular neutral-alkaline pH—is most active at a narrow, more acidic range of pH values (pH 5–6), specifically toward the C-26 position of GAA.

A few reports demonstrated that triterpenoid glycosides may improve the bioactivity of the triterpenoid aglycone [5]. Liang et al. produced an unusual ginsenoside, 3β, 12β-di-*O*-Glc-protopanaxadiol (PPD), from the glucosylation of PPD by UGT109A1, and showed that the ginsenoside had anti-cancer capabilities in the Lewis lung cancer xenograft mouse model [23]. Wang et al. used BsGT1 to produce 3β-*O*-Glc-ginsenoside F1, which inhibited melanin and tyrosinase activities [25]. Dai et al. reported the enzymatic synthesis of glycyrrhetinic acid (GA) glucosides—GA-30-*O*-β-glucoside and GA-3-*O-*β-glucoside—by BsYjiC and found that the two triterpenoid glucosides had higher water solubility and higher cytotoxicity against human liver cancer cells HepG2 and breast cancer cells MCF-7 than GA aglycone [20]. Therefore, the new GAA glucoside obtained in the present study, GAA-26-*O*-β-glucoside, warrants future investigation to determine whether it also has a higher bioactivity than GAA aglycone.

In summary, even though over 300 triterpenoids have been found in *G. lucidum*, very few triterpenoid glycosides have been identified [4]. Our study was the first to reveal that a single bacterium, the *Bacillus subtilis* ATCC 6633 strain, can biotransform GAA into both GAA-26-*O*-β-glucoside by BsGT110 in specific acidic conditions and GAA-15-*O*-β-glucoside by BsGT398 and BsGT489 in neutral-alkaline conditions.

## 4. Materials and Methods 

### 4.1. Chemicals and Recombinant Enzymes

GAA was purchased from Baoji Herbest Bio-Tech (Xi-An, Shaanxi, China). UDP-glucose was obtained from Cayman Chemical (Ann Arbor, MI, USA). Recombinant BsGT enzymes (BsGT110, BsUGT398, BsUGT489, BsGT292, and BsGT296) were obtained from our previous studies [6,17]. The other reagents and solvents used were commercially available. 

### 4.2. Glycosylation of GAA by Recombinant Enzymes

Glycosylation was performed in 0.1 mL reaction mixture containing 1 mg/mL GAA, 15 μg/mL the recombinant enzymes, 10 mM MgCl_2_, and 10 mM UDP-glucose at pH 5-6 (50 mM acetate buffer) or pH 6–8 (50 mM PB). The reaction was performed at 40 °C for 30 min. Afterward, the reaction mixture was analyzed with UPLC. For optimization experiments, the above reaction mixture was incubated with 50 mM acetate buffer (pH 6) at different temperatures or with different metal ions.

For the kinetic experiments, different concentrations of GAA were mixture with 15 μg purified BsGT110 protein, 10 mM UDP-glucose, 10 mM MgCl_2_, and 50 mM PB (pH 7.0) or acetate buffer (pH 6) in 1 mL reaction mixture and incubated at 40 °C for 20 min. During the incubation, samples from each reaction were removed every 2 min and analyzed by UPLC. The amount of GAA-26-*O*-β-glucoside production from the reaction was calculated from the peak area of UPLC analysis normalized with a standard curve. The rate of the reaction at each concentration of GAA was obtained from the slope of the plot of the amount of product over time. Kinetic parameters were obtained from the double-reciprocal plot of substrate GAA concentration versus the rate of reaction. 

### 4.3. Ultra-Performance Liquid Chromatography (UPLC)

UPLC was performed with an Acquity^®^ UPLC system (Waters, Milford, MA, USA). The stationary phase was a C18 column (Acquity UPLC BEH C18, 1.7 μm, 2.1 i.d. × 100 mm, Waters, MA, USA), and the mobile phase was 1% acetic acid in water (A) and methanol (B). The linear gradient elution condition was 0 min with 36% B to 7 min with 81% B at a flow rate of 0.2 mL/min. The detection condition was set at 254 nm.

### 4.4. Purification and Identification of the Glycosylated Product

Twenty-five mL of the reaction mixture (1 mg/mL GAA, 15 μg/mL BsGT110, 10 mM UDP-glucose, 10 mM MgCl_2_, 50 mM acetate buffer at pH 6) was carried out at 40 °C for 30 min. Afterward, an equal volume of methanol was added into the reaction mixture to stop the reaction. Fifty mL of the reaction mixture with 50% methanol was applied to a preparative YL9100 HPLC system (YoungLin, Gyeonggi-do, Korea). The stationary phase was the Inertsil ODS 3 column (10 mm, 20 i.d. × 250 mm, GL Sciences, Eindhoven, The Netherlands), and the mobile phase was the same as that in the UPLC system, but with a flow rate of 15 mL/min. The detection condition was 254 nm, and the sample volume was 10 mL for each injection. The product of each run was collected, concentrated under a vacuum, and lyophilized with a freeze dryer. From the 25 mL of reaction, 5.4 mg of the product was purified. The chemical structure of the product compound was determined with mass and NMR spectral analyses. The mass spectral analysis was performed on a Finnigan LCQ Duo mass spectrometer (ThermoQuest Corp., San Jose, CA, USA) with ESI. ^1^H- and ^13^C-NMR, HSQC, and HMBC spectra were recorded on a Bruker AV-600 NMR spectrometer (Bruker Corp., Billerica, MA, USA) at ambient temperature. Standard pulse sequences and parameters were used for the NMR experiments, and all chemical shifts were reported in parts per million (ppm, δ). 

## 5. Conclusions

A new GAA-26-*O*-β-glucoside was produced from the *O*-glucosylation of GAA with recombinant BsGT110 isolated from *B. subtilis* ATCC 6633 under acidic conditions (pH 6). BsGT110 was the first GT identified as catalyzing the glycosylation of triterpenoid at the C-26 position. Moreover, the optimal reaction condition of BsGT110 was at pH 6, and it lost most of this activity at pH 8, implying that such GTs might only catalyze other triterpenoid substrates under acidic conditions.

## Figures and Tables

**Table 1:** Relative production ^a^ of GAA-15-*O*-β-glucoside and compound (**2**) catalyzed from GAA by BsGT110.

pH Value	Production of GAA-15-*O*-β-glucoside	Production of Compound (2)
5 ^b^	1.13 ± 0.13	87.96 ± 4.67
6 ^b^	4.31 ± 0.27	100.00 ± 7.85 ^a^
6 ^c^	4.85 ± 0.15	84.53 ± 9.49
7 ^c^	15.26 ± 0.64	30.81 ± 0.71
8 ^c^	34.02 ± 0.94	16.04 ± 1.04

^a^ Relative production was normalized to the UPLC area of the peak of compound (**2**) in an acetate buffer of pH 6. ^b^ 50 mM of acetate buffer. ^c^ 50 mM of PB.

**Table 2:** Kinetic parameters of BsGT110 toward GAA at pH 6 and pH 7.

Reaction Condition	K_m_ (μM)	k_cat_ (min^−1^)	k_cat_ /K_m_ (min^−1^μM^−1^)
pH 6	570.6 ± 29.4	11.2 ± 0.9	0.0196 ± 0.0007
pH 7	299.4 ± 84.4	3.8 ± 0.9	0.0149 ± 0.0074

## Supplementary Materials

The following are available online. Table S1. NMR spectroscopic data for compound (**2**) in pyridine-d_5_ (600 MHz), Table S2. BsGT110 sequence comparison with candidate triterpenoid-catalyzing GTs and flavonoid-catalyzing GTs, Figure S1. The ^1^H-NMR (600 MHz) spectrum of compound (**2**) in pyridine-d_5_, Figure S2. The ^13^C-NMR (150 MHz) spectrum of compound (**2**) in pyridine-d_5_, Figure S3. The HSQC (600 MHz) spectrum of compound (**2**) in pyridine-d_5_, Figure S4. The HMBC (600 MHz) spectrum of compound (**2**) in pyridine-d_5_, Figure S5. Phylogenetic analysis using the Maximum Likelihood method.
