# Supplementary material for: A New Triterpenoid Glucoside from a Novel Acidic Glycosylation of Ganoderic Acid A via Recombinant Glycosyltransferase of Bacillus subtilis
Source: Molecules. 2019 Sep 24;24(19):3457. doi: 10.3390/molecules24193457 (PMC6804120; doi:10.3390/molecules24193457)
Supplement: Supplementary file 1 [file molecules-24-03457-s001.pdf]

Supplementary Materials: A New Triterpenoid Glucoside from a Novel Acidic Glycosylation of Ganoderic Acid A via Recombinant Glycosyltransferase of *Bacillus subtilis*

Te-Sheng Chang [1,+], Chien-Min Chiang [2,+], Yu-Han Kao [1], Jiumn-Yih Wu [3], Yu-Wei Wu [4,5] and Tzi-Yuan Wang [6,*]

[1] Department of Biological Sciences and Technology, National University of Tainan, Tainan 70005, Taiwan; mozyme2001@gmail.com (T.-S.C.); aa0920281529@gmail.com (Y-H.K.)
[2] Department of Biotechnology, Chia Nan University of Pharmacy and Science, No. 60, Sec. 1, Erh-Jen Rd., Jen-Te District, Tainan 71710, Taiwan; cmchiang@mail.cnu.edu.tw
[3] Department of Food Science, National Quemoy University, Kinmen County 892, Taiwan; wujy@nqu.edu.tw
[4] Graduate Institute of Biomedical Informatics, College of Medical Science and Technology, Taipei Medical University, Taipei 106, Taiwan; yuwei.wu@tmu.edu.tw
[5] Clinical Big Data Research Center, Taipei Medical University Hospital, Taipei 110, Taiwan;
[6] Biodiversity Research Center, Academia Sinica, Taipei 115, Taiwan; tziyuan@gmail.com

[*] Correspondence: tziyuan@gmail.com (T.-Y.W.); Tel.: +886-2-27872258 (T.-Y.W.); Fax: +886-2-27899624 (T.-Y.W.)
[+] The two authors contributed equally

Table S1. NMR sprctroscopic data for compound (**2**) in pyridine-d_5 (600MHz)

Compound	GAA-26-O-β-glucoside Compound (**2**)			
Position	δ_C ,	type	δ_H (*J* in Hz)	HMBC
GAA moiety				
1	35.7	CH_2	1.57, m; 3.08, m	H-2, H-19
2	34.2	CH_2	2.54, m 2.60, m	H-1
3	215.8	C		H-1, H-2, H-28, H-29
4	46.4	C		H-6, H-28, H-29
5	48.6	CH	1.83, m	H-1, H-6, H-19, H-29
6	29.3	CH_2	1.96, m; 2.24, m	H-5, H-7
7	68.3	CH	4.93, t (8.8)	H-5, H-6
8	161.1	C		H-6, H-7, H-15, H-30
9	139.6	C		H-1, H-7, H-12, H-19
10	37.9	C		H-1, H-2, H-5, H-6, H-19
11	199.3	C		H-12,
12	52.0	CH_2	2.64, d (15.8); 2.92, d (15.8)	H-18
13	46.7	C		H-12, H-17, H-18, H-30
14	54.3	C		H-12, H-15, H-16, H-18, H-30
15	71.8	CH	5.22, t (8.3)	H-30,
16	36.5	CH_2	1.89, m; 2.12, m	H-15, H-17
17	48.2	CH	1.88, m	H-16, H-18, H-21, H-22
18	17.1	CH_3	1.06, s	H-12, H-17
19	19.2	CH_3	1.41, s	H-1,
20	32.6	CH	2.13, m	H-16, H-17, H-21, H-22,
21	19.3	CH	0.90, t (6.5)	H-22
22	49.5	CH_2	2.23, m; 2.49, m	H-21
23	207.8	C		H-22, H-24, H-25
24	46.1	CH_2	2.55, m ; 2.99, m	H-25, H-27
25	34.8	CH	3.23, m	H-24, H-27
26	174.6	C		H-24, H-25, H-27, **Glc-H-1′**
27	16.4	CH_3	1.19, d (7.3)	H-24, H-25
28	26.9	CH_3	1.15, s	H-5, H-29
29	20.5	CH_3	1.11, s	H-5, H-28
30	19.9	CH_3	1.50, s	H-7, H-15, H-16
Glucose moiety				
1′	95.9		6.33, d (8.1)	H-2′, H-3′
2′	73.9		4.20, m	H-3′
3′	78.2		4.29, m	H-2′
4′	70.6		4.35, m	H-3′, H-6′
5′	79.1		4.03, m	H-1′, H-6′
6′	61.8	CH_2	4.39, m ; 4.46, m	H-4′

Table S2. BsGT110 sequence comparison with candidate triterpenoid-catalyzing GTs and flavonoid-catalyzing GTs.

Candidate GTs	E-value	Score	Hit start	Hit end	Hit length	**%Identity**	%Gaps
BsYjiC_(NP_389104)	1.36E-63	515	5	364	360	**31.83**	5.57
BsGT1_(ANP92054)	1.36E-63	515	5	364	360	**31.83**	5.57
UGT109A1_(ASY97769)	2.41E-61	500	5	351	347	**31.59**	5.77
BsUGT489_(WP_003220489)	2.41E-61	500	5	351	347	**31.59**	5.77
BsUGT398_(WP_003225398)	3.09E-77	608	1	369	369	**33.87**	1.6
**BsGT110_(WP_003220110)***	**0**	**2065**	**1**	**405**	**405**	**100**	**0**
BlYjiC_(AAU40842)	3.67E-60	492	5	369	365	**30.13**	3.2
BcGT1_(AAS41089)	1.46E-103	786	1	373	373	**40.69**	1.06
BcGT3_(AAS41737)	4.69E-88	682	1	371	371	**36.8**	1.87
OleD_(ABA42119)	1.42E-48	412	8	377	370	**26.87**	7.75
XcGT2_(AAM41712)	6.68E-08	100	202	348	147	**28.21**	8.97

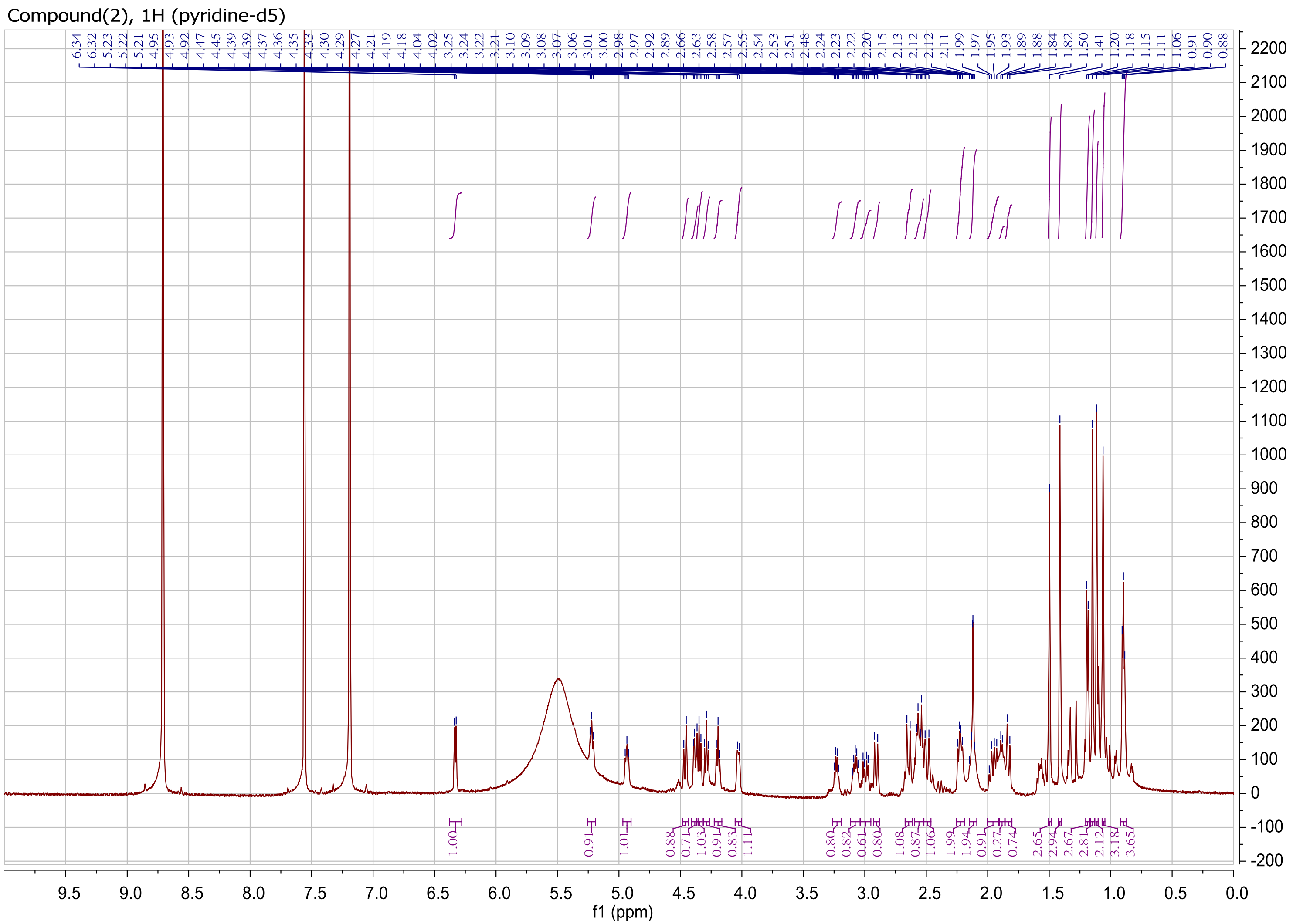

Figure S1. The ^{1}H-NMR (600 MHz) spectrum of compound (**2**) in pyridine-d_5.

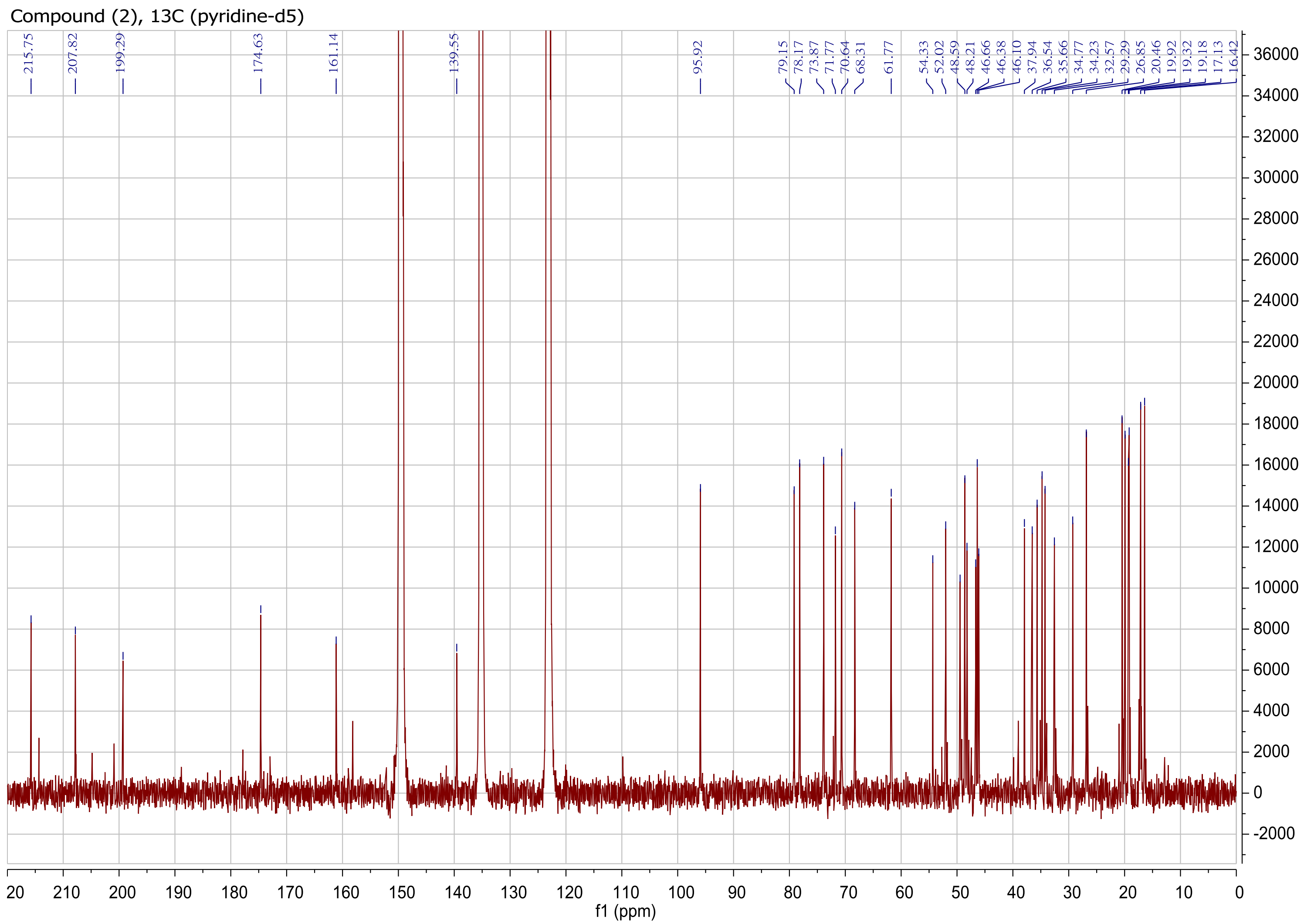

Figure S2. The ^{13}C-NMR (150 MHz) spectrum of compound (**2**) in pyridine-*d*$_5$.

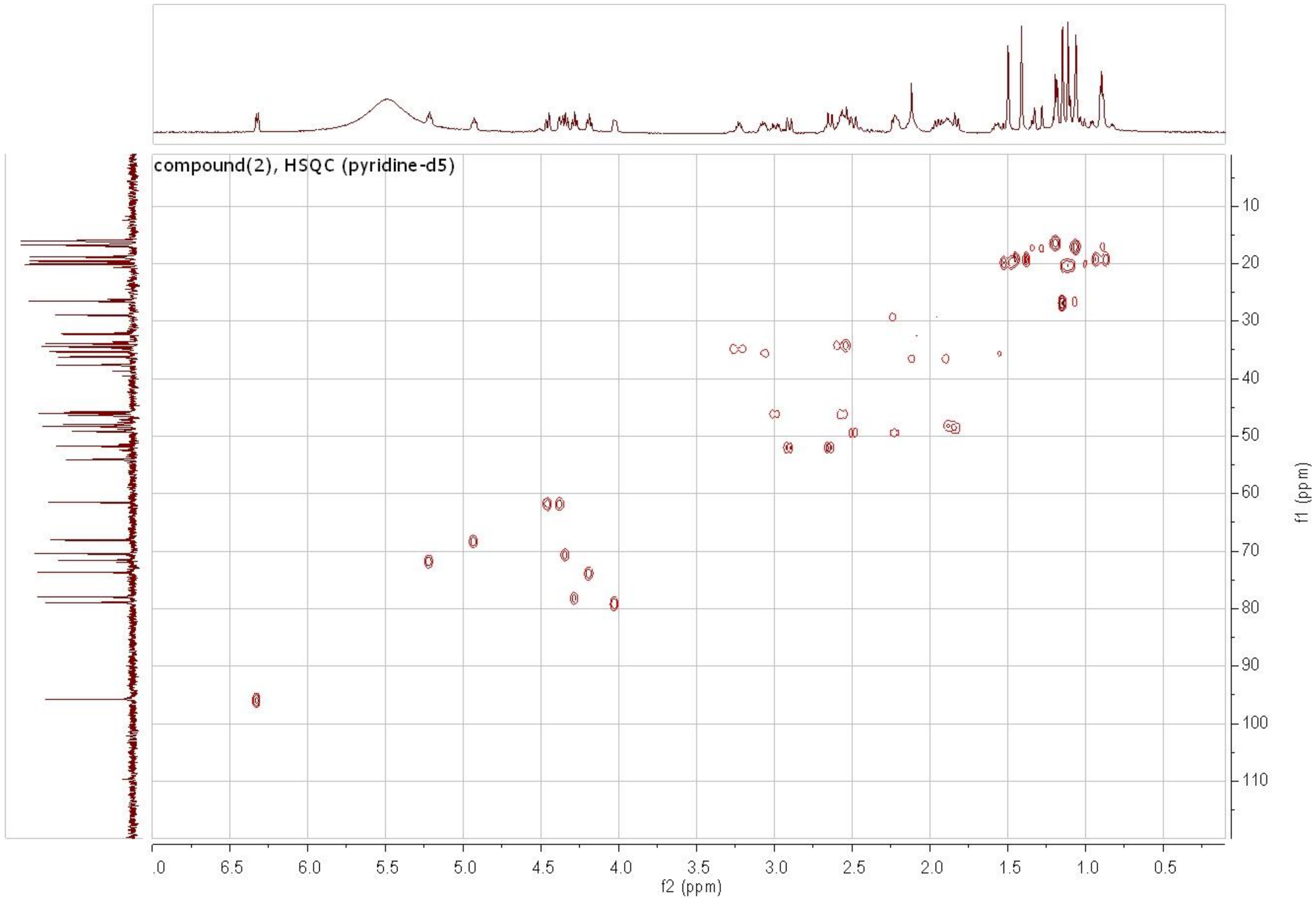

Figure S3. The HSQC (600 MHz) spectrum of compound (**2**) in pyridine-d_5.

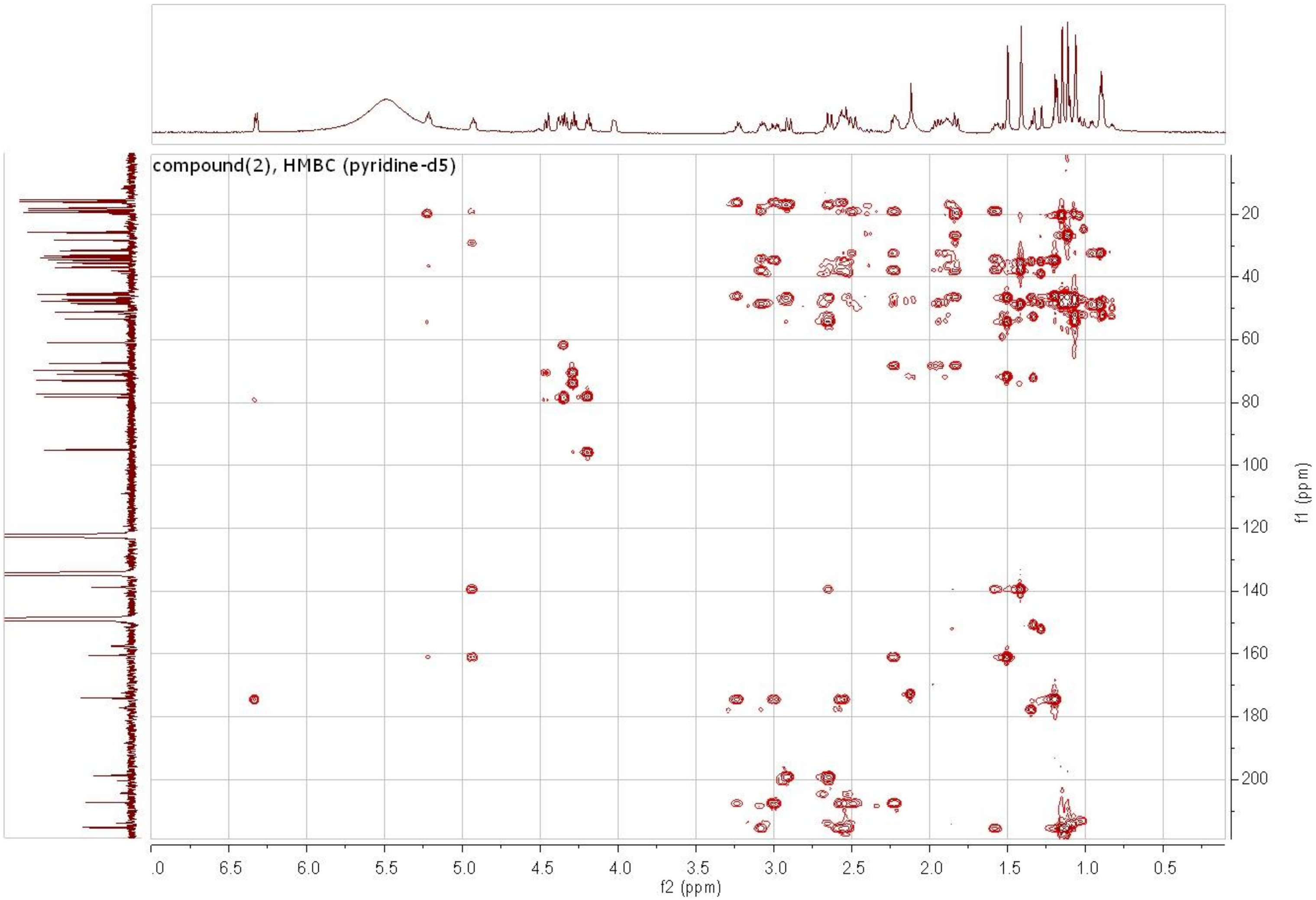

Figure S4. The HMBC (600 MHz) spectrum of compound (**2**) in pyridine-*d*5.

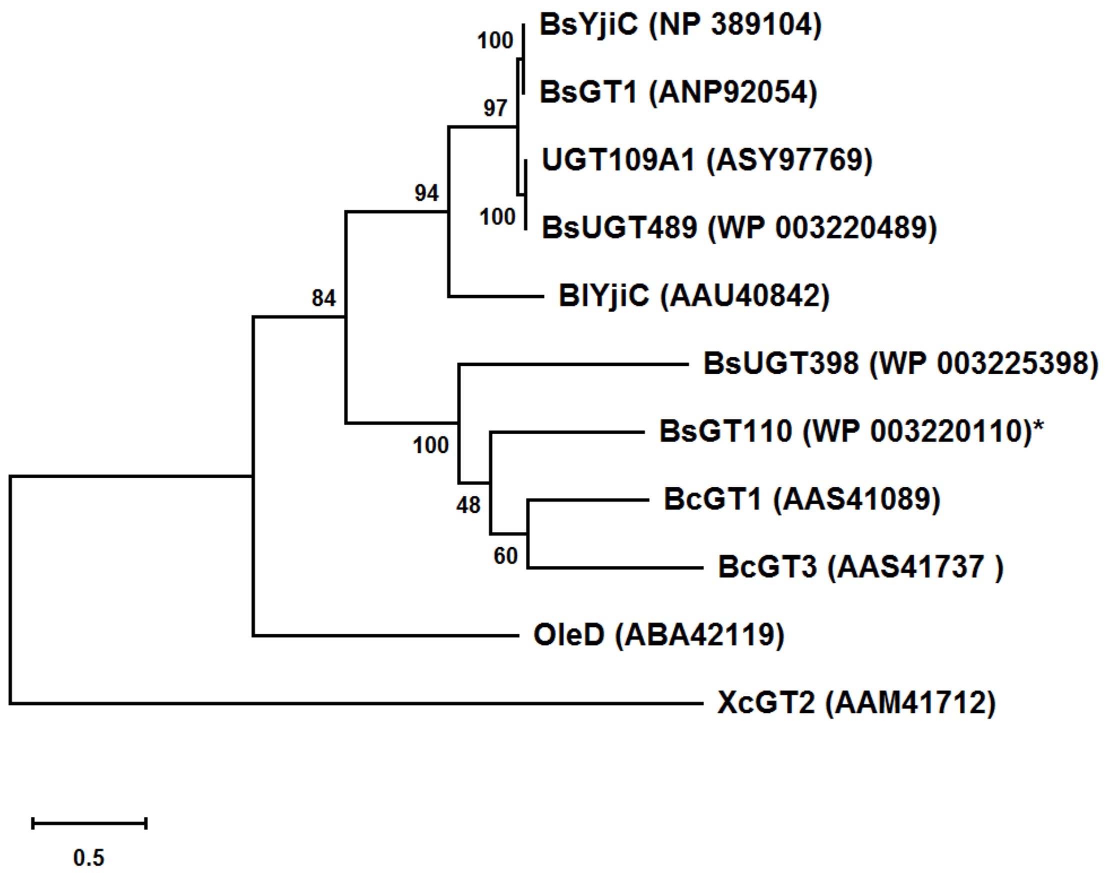

Figure S5. Phylogenetic analysis using the Maximum Likelihood method. The evolutionary history was inferred by using the Maximum Likelihood method and General Reversible Mitochondrial model. The tree with the highest log likelihood (-7192.36) is shown. The percentage of trees in which the associated taxa clustered together is shown next to the branches. Initial tree(s) for the heuristic search were obtained automatically by applying Neighbor-Join and BioNJ algorithms to a matrix of pairwise distances estimated using a JTT model, and then selecting the topology with superior log likelihood value. The tree is drawn to scale, with branch lengths measured in the number of substitutions per site. This analysis involved 11 amino acid sequences. All positions with less than 95% site coverage were eliminated, i.e., fewer than 5% alignment gaps, missing data, and ambiguous bases were allowed at any position (partial deletion option). There were a total of 368 positions in the final dataset. Evolutionary analyses were conducted in MEGA X.

© 2017 by the authors. Submitted for possible open access publication under the terms and conditions of the Creative Commons Attribution (CC BY) license (http://creativecommons.org/licenses/by/4.0/).
